# Establishment of a clear cell renal cell carcinoma organoid cohort integrated into the UroCCR clinical database: A feasibility study

**DOI:** 10.1016/j.tranon.2026.102885

**Published:** 2026-06-29

**Authors:** R. Lefranc, T. Waeckel, M. Riffet, R. Florent, G. Desmartin, L. Lecouflet, J. Divoux, L. Poulain, X. Tillou, L.B. Weiswald, G. Levallet, C. Bazille

**Affiliations:** a Université de Caen Normandie, CNRS, Normandie Univ, ISTCT UMR6030, CYCERON, F-14000 Caen, France; b Centre Hospitalier Universitaire de Caen Normandie, Service d’Urologie et Transplantation, 14000 Caen, France; c Centre Hospitalier Universitaire de Caen Normandie, Service d’Anatomopathologie, 14000 Caen, France; d Université de Caen Normandie, PLATON Services Unit, ORGAPRED core facility, Caen, France; e UNICANCER, Comprehensive Cancer Center François Baclesse, Caen, France; f Université de Caen Normandie, INSERM U1086 ANTICIPE (Interdisciplinary Research Unit for Cancers Prevention and Treatment), BioTICLA laboratory (Precision medicine for ovarian cancers), Caen, France

**Keywords:** Preclinical model, Organoids, Personalized medicine, Clear cell carcinoma, UroCCR network

## Abstract

**Background:**

Clear cell renal cell carcinoma (ccRCC) is the sixth most common cancer in France. While early-stage survival rates are high in early stages, they decrease significantly in metastatic stages, due to tumor heterogeneity and treatment resistance. The development of reliable preclinical models is essential to improve targeted therapies. This study aimed to evaluate the feasibility of establishing patient-derived ccRCC organoids linked to the UroCCR clinical database.

**Methodology:**

Tumor samples from total or partial nephrectomies performed at Caen University Hospital between November 2023 and March 2024 were processed for organoid culture. Seven culture conditions were tested, including different extracellular matrices (Matrigel, collagen I) and growth factor–enriched media.

**Results:**

Organoids were established in 12 of 18 cases (66.7%). Among these, five cultures were successfully expanded and characterized histologically (42%). Organoids were maintained in culture for a median of 52 days and underwent a mean of two passages. Histological analyses showed that organoids retained several morphological features consistent with the original tumors, although variability in CA-IX and CK7 expression was observed.

**Significance:**

This feasibility study demonstrates that ccRCC organoid generation from surgical specimens is achievable but remains limited by variable establishment rates and culture duration. Further optimization and integration of microenvironmental components will be necessary to enhance the translational relevance of this model

## Highlights

• **Clinical Integration**: ccRCC organoids linked to UroCCR database (NCT03293563).
• **Methodology**: 42% establishment rate with optimized culture protocols.
• **Authenticity**: Organoids preserve tumor morphology and molecular markers (CA-IX, CK7).
• **Sustainability**: 52-day median culture duration for therapeutic screening.
• **Translation**: Platform bridges laboratory research with precision oncology.

## Introduction

In France, kidney cancer is the sixth most common cancer, with approximately 15,300 new cases and over 5500 deaths annually. Clear cell renal cell carcinoma (ccRCC) is the predominant histological subtype, accounting for nearly 75% of cases [1]. While early-stage or locally advanced ccRCC has a favorable prognosis, with a five-year survival rate of 90%, about 30% of patients develop metastatic disease, where survival drops dramatically to 10%, despite advancements in targeted therapies and immunotherapy [2]. To date, metastasis treatment is based on a combination of dual immunotherapy (IO – IO) or immunotherapy combined with a tyrosine kinase inhibitor (IO – ITK) [3]. One of the major challenges in ccRCC treatment is the 25% non-response rate to first-line therapies, attributed to significant tumor heterogeneity [4]. Unfortunately, no predictive or theragnostic biomarker exists.in ccRCC.

These limitations highlight the need for improved therapeutic strategies, either through the development of more effective targeted treatments or the optimization of existing ones [5]. Personalized medicine, which tailors’ treatments based on individual tumor characteristics, is emerging as a promising approach [6,7]. Progress in this field is hindered by the lack of reliable preclinical models capable of accurately reproducing ccRCC biology and predicting treatment response. Although there are several known ccRCC cell lines, such as 786–0, traditional *vitro* 2D cell cultures are limited by flat, non-physiological environments, limiting their clinical relevance. In addition to ethical and cost issues, the murine xenograft model, long used to study TKi in ccRCC, poses problems for studying IO. In contrast, Patient-Derived Tumor Organoids (PDTO) have been shown to better reproduce tumor morphology, heterogeneity, and response to therapy [8].

While PDTO models have been developed for several cancers, such as head and neck squamous cell carcinoma and ovarian cancers [9], and series of studies are now exploring the use of this model in clinical situations [10,11], the field of ccRCC PDTO remains underexplored. To date, only few studies have reported the feasibility of establishing ccRCC PDTO remains technically challenging, with variable establishment rates reported across studies [[12], [13], [14]]. Furthermore, most published protocols focus primarily on culture feasibility and characterization, and integration with structured clinical data remains limited and have not been integrated into a biobank associated with clinical data.

The present study aimed to evaluate the feasibility of generating patient-derived ccRCC organoids from surgical specimens within the UroCCR framework, a French multicenter clinical database, and to characterize their initial morphological and immunohistochemical features.

## Methods

### Patient inclusion and sample collection

**Fig. 1:**
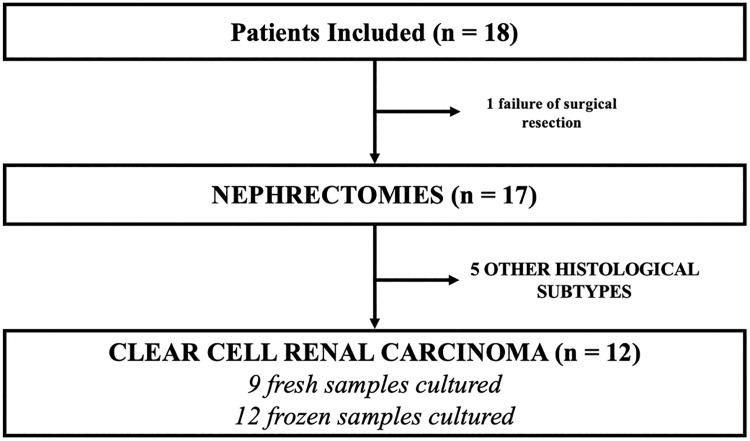
Flow chart.

Renal tumor samples were collected from patients undergoing radical or partial nephrectomy at Caen University Hospital between November 2023, and March 2024. Inclusion criteria consisted of adults (>18 years) diagnosed with ccRCC. Exclusion criteria included tumors <4 cm (cT1a), cystic lesions, prior immunotherapy and/or tyrosine kinase inhibitor, and non-ccRCC histology (Fig. 1). Tumors measuring <4 cm in diameter were excluded because limited tissue volume was insufficient to allow parallel sampling for histology, biobanking, and organoid culture.

Written informed consent was obtained from all patients in accordance with the French Data Protection Law (January 6, 1978), the General Data Protection Regulation (EU No 2016/679), and French Law No 2018–493 (June 20, 2018). The study complied with Public Health Law No 2004–806 and its implementation decrees. Ethical approval was granted by the Local Ethics and Health Research Committee (CLERS) of Caen University Hospital on August 30, 2023 (Application No 4743). The UroCCR project (NCT03293563) has been approved by the Advisory Committee on the Processing of Health Research Information (CCTIRS) and received French Data Protection Authority (CNIL) authorization (DR-2013–206, DR-2016–485, and deliberation No 2022–091).

### Tumor sample processing and organoid culture

Tumor specimens were immediately transported unfixed to the pathology laboratory. Three samples were allocated for PDTO culture, three mirror samples for histology, and two samples to the tumor library (INNOVABIO). Tissue fragments were taken away from tumor margins to avoid contamination with healthy tissue.

PDTO samples were placed in transport medium (Advanced DMEM - *GIBCO™ Thermo Fisher Scientific, Waltham, MA, USA*, GlutaMAX-1 - *GIBCO™ Thermo Fisher Scientific, Waltham, MA, USA*, Penicillin/Streptomycin - *GIBCO™ Thermo Fisher Scientific, Waltham, MA, USA*, Y-27,632 dihydrochloride - *Interchim SA, Montluçon, France*, Caspofungin - *Sigma-Aldrich, Saint-Louis, MO, USA*) pre-cooled at 4 °C. The remainder of the specimen was fixed in paraformaldehyde for paraffin embedding, cryopreserved in freezing solution (10% DMSO, 90% non-inactivated FBS) for future isolation of viable cells or snap frozen for molecular analyses.

### Tissue dissociation and PDTO establishment

Tumor fragments were minced (1–2 mm³) and enzymatically digested using the MACS® Tissue Dissociation Kit (Miltenyi Biotec) in RPMI medium with Penicillin/Streptomycin. The suspension was processed in the GentleMACS OctoDissociator® using a dissociation program tailored to the tumor consistency (Soft, Medium, Tough).

Cells were filtered (200 µm filter), washed, and centrifuged (430 g, 5 min, 4 °C). Cells were counted and resuspended in culture medium and extracellular matrix (Matrigel, BME2 or Collagen I). Cell droplets (10,000–15,000 cells, 50 µL) were seeded in 24-well plates and incubated at 37 °C. The culture medium was refreshed twice a week.

### PDTO passaging and maintenance

Passaging was performed when the PDTO reached optimal growth. Extracellular matrix was mechanically disrupted by repeated pipetting in cold OBM-BSA, and PDTO were dissociated by enzymatic digestion using TrypLE™ Express (37 °C, 5–15 min). Cells were resuspended in fresh medium, counted, and reseeded. Organoids were cryopreserved in freezing solution (Recovery™ Cell Culture Freezing Medium) and stocked at −150 °C as necessary.

### Air-Liquid interface (ALI) culture

For ALI conditions, type I collagen (Rat Tail) was mixed with Medium 199, NaOH, and distilled water to reach pH 6.5–7.5. MILLICELL® culture inserts (12 mm, 4 µm pores) were coated with 400 µL of collagen and seeded with an equal volume of tumor cells. The inserts were placed in wells containing 600 µL of medium and maintained at 37 °C.

Passaging involved digestion with type IV collagenase (200 U/mL, 37 °C, 30 min), followed by centrifugation and reseeding. Medium changes were performed twice a week.

### PDTO characterization

PDTO were harvested, washed with PBS, and fixed in 3% PFA overnight at 4 °C. Samples were embedded in agarose and processed for paraffin embedding. Sections (3 µm) were stained with H&E and analyzed by immunohistochemistry using carbonic anhydrase 9 - CA-IX (EP 161- Diagomics – Reference BSB-6415 - pre-diluted) and cytokeratin 7 - CK7 (OV-TL12/30 - Dako – Reference M7018 – dilution: 1/200) antibodies, two markers characteristic of ccRCC (CA-IX positive and CK7 negative) on a Ventana Benchmark ULTRA automated system. Images shown were obtained using an S360 NanoZoomer® scanner (Hamamatsu).

### Statistical analysis

GraphPad Prism version 10.4.2 (GraphPad Software, San Diego, CA, USA) was used for descriptive statistics and analyze culture success rates and viability.

## Results

### Patient and tumor characteristics

A total of 18 patients were included in the study, with a median age of 68 years (range: 56–76 years) and a male-to-female ratio of 1.6. Seventeen patients underwent nephrectomy, including nine total nephrectomies (53%) and eight partial nephrectomies (47%).

Final histopathological confirmation of ccRCC in 12 patients (70.0%) and subsequent organoid analyses were restricted to these confirmed cases. The median tumor stage was pT1b, and the median ISUP/OMS grade was 2. At diagnosis, 16.7% (n = 3) of patients presented with metastases. Details are presented in Table 1.

**Table 1:** Characteristics of the population.

CASE	AGE	SEX	SURGERY	HISTOLOGY	T Stage	N Stage	M Stage	ISUP
R23.001	59	M	Partial	ccRCC	pT1b	0	0	2
R23.002	59	F	Total	ccRCC	pT3a	0	1	2
R23.003	71	M	Total	ccRCC	pT1a	0	0	2
R24.001	64	F	Total	Chromophobe	pT1b	0	0	NA
R24.002	68	M	Partial	ccRCC	pT1a	0	0	2
R24.003	75	M	Partial	Papillary	pT1a	0	0	NA
R24.004	77	M	Partial	ccRCC	pT1a	0	0	2
R24.005	54	M	FAILURE	NA	NA	NA	NA	NA
R24.006	76	H	Partial	Oncocytoma	pT1a	0	0	NA
R24.007	67	F	Total	ccRCC	pT3c	0	1	4
R24.008	77	F	Total	ccRCC	pT3a	0	1	1
R24.009	54	M	Total	Chromophobe	pT3a	0	0	NA
R24.010	46	F	Partial	ccRCC	pT2a	0	0	2
R24.011	41	F	Total	ccRCC	pT1b	0	0	2
R24.012	84	F	Total	ccRCC	pT2b	0	0	3
R24.013	41	M	Total	ccRCC	pT3a	0	0	2
R24.014	70	M	Partial	ccRCC	pT1b	0	0	2
R24.015	77	M	Partial	Chromophobe	pT1a	0	0	NA

### ccRCC PDTO culture

We explored seven different culture conditions designed to optimize organoid establishment and growth (Table 1). Conditions varied along three main axes: (i) basal medium composition (Advanced DMEM versus Advanced DMEM/F12), as nutrient formulations may differentially support epithelial cell survival; (ii) extracellular matrix type (Matrigel versus type I collagen), given their distinct mechanical and biochemical properties affecting three-dimensional organization; and (iii) growth factor supplementation, including EGF, FGF, and B27, selected based on their known roles in supporting renal epithelial proliferation. Conditions 5 and 6 were specifically designed for cryopreserved samples, incorporating extended Y-27,632 exposure during the first seven days to enhance post-thaw cell viability. Condition 7 used an air–liquid interface approach, previously reported to better preserve tumor microenvironment components. However, Y-27,632 continued to be added during enzymatic dissociation and passaging to limit anoïkis. The components for each condition are detailed in Table 2.

**Table 2:** Organoid medium components.

	CELL CULTURE CONDITION						
	No 1	No 2	No 3	No 4	No 5	No 6	No 7
BASAL MEDIUM							
OBM*	43.7 mL	48.7 mL	48.7 mL	43.7 mL	48.8 mL	-	18.2 mL
OBM + F12⁎⁎	-	-	-	-	-	47.7 mL	-
MEDIUM COMPONENTS							
A8301 (3 µM)	50 µL	-	-	50 µL	-	-	50 µL
B27 Supplement (1X)	1 mL	1 mL	1 mL	1 mL	1 mL	1 mL	1 mL
EGF (50 ng/mL)	5 µL	5 µL	5 µL	5 µL	5 µL	5 µL	5 µL
FGF-b (20 ng/mL)	-	100 µL	100 µL	-	-	-	-
FGF-10 (100 ng/mL)	50 µL	-	-	50 µL	-	-	-
Heparin (4 µg/mL)	-	100 µL	100 µL	-	-	-	-
HEPES (10 nM)	-	-	-	-	-	500 µL	-
IL-2 (600 mUI/mL)	-	-	-	-	-	-	5 µL
Leu-Gastrin (10 µM)	-	-	-	-	-	-	5 µL
L-WRN (50%)	-	-	-	-	-	-	25 mL
N-acetylcysteine (1.25 mM)	100 µL	-	-	100 µL	-	100 µL	100 µL
Nicotinamide (10 nM)	500 µL	-	-	500 µL	-	500 µL	500 µL
Noggin (25 ng/mL)	-	-	-	-	-	-	12.5 µL
PRIMOCINТМ (100 ng/mL)	100 µL	100 µL	100 µL	100 µL	100 µL	100 µL	100 µL
R-Spondin (10%)	5 mL	-	-	5 mL	-	-	-
SB202190 (10 µM)	-	-	-	-	16.7 µL	16.7 µL	1.67 µL
Y27632 (10 µM)	50 µL	50 µL	50 µL	50 µL	50 µL	50 µL	50 µL
EXTRACELLULAR MATRIX							
BME 2	X	-	X	-	X	X	-
MATRIGEL®	-	X	-	X	-	-	-
Rat Tail Type 1 Collagen + MILLICELL® standing cell culture insert 0.4 µm, 12 mm	-	-	-	-	-	-	X
(air-liquid interface)							

⁎ OBM (organoid basis medium): 500 mL Advanced DMEM + 5 mL GlutaMAX (100X) + 13,7 mL Penicillin/Streptomycine (100X).

⁎⁎ OBM + F12: 250 mL Advanced DMEM + 250 mL Ham's F12 + 5 mL GlutaMAX (100X) + 13,7 mL Penicillin/Streptomycine (100X).

PDTO were successfully established in 12 out of 18 cases (66.7%). Nine cultures were initiated immediately after surgery, while three were derived from cryopreserved tumor samples. Across all conditions, PDTO persisted in culture for a median of 52 days (range: 44–67 days) and underwent an average of two passages per culture.

Among the seven culture conditions tested, five supported PDTO growth, with condition No 2 yielding optimal results in 5/12 cases (41.7%). This condition contained EGF, FGF, heparin, and the B27 supplement. Conditions No 1, No 5, and No 6 were effective in two cases each (16.7%), while condition No 3 supported viable cultures in only one case (8.3%). Conditions No 4 and No 7 failed to sustain growth over a few days. Organoids cultured under ALI conditions (Condition 7) failed to demonstrate sustained growth beyond the first week. Several technical limitations were encountered: (i) the opacity of the type I collagen matrix prevented reliable monitoring of organoid formation and growth using standard phase-contrast microscopy (Table 3); (ii) the collagen gel consistency made passaging difficult, with frequent mechanical disruption of forming structures during collagenase digestion; and (iii) the limited number of cells available per sample, combined with the requirement for parallel testing of multiple conditions, reduced the cellular input allocated to ALI cultures. Given these constraints, ALI cultures were discontinued early in the study.

**Table 3:** Organoid culture results.

CASE	NUMBER of CULTURE CONDITIONS USED	CULTURE on FRESH SAMPLE	CULTURE on FROZEN SAMPLE	BEST CONDITION*	DAYS of CULTURE	PASSAGES	CHARACTERIZATION
R23.001	3	+	+	1	75	3	NO
R23.002	2	-	+	6	55	2	NO
R23.003	7	+	+	1	95	4	YES
R24.002	5	+	+	2	52	3	NO
R24.004	5	+	+	5	79	3	YES
R24.007	2	-	+	6	63	2	YES
R24.008	5	+	+	2	44	2	NO
R24.010	6	+	+	2	45	2	NO
R24.011	6	+	+	2	41	1	NO
R24.012	6	+	+	5	67	3	YES
R24.013	2	-	+	5	14	0	NO
R24.014	6	+	+	3	28	1	YES

⁎ The best culture condition was defined as the one that allowed the highest number of passages, thereby maintaining the culture viable for the longest time.

### Morphological features of ccRCC organoids

**Fig. 2:**
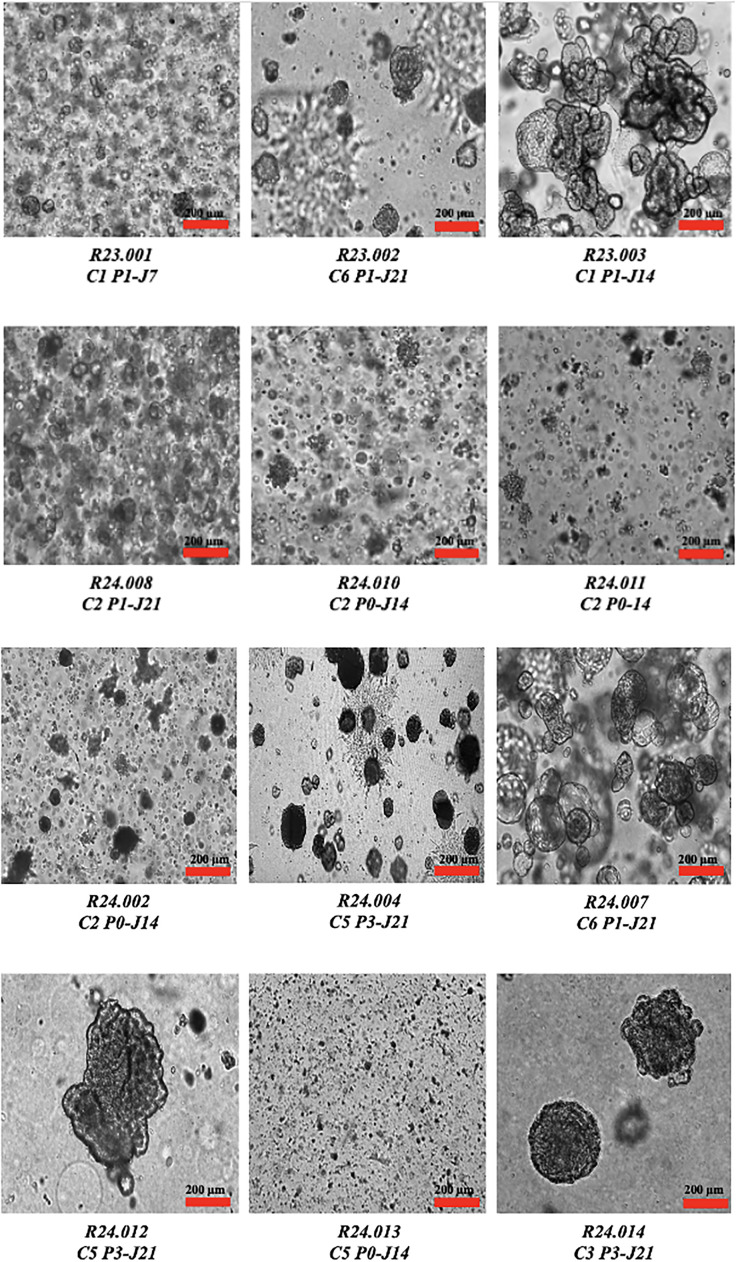
PDTO under phase contrast microscope. *All cultures are presented under microscopy at 4x magnification. Scale is 200 µm. **R23003 and R24.007: These samples exhibit two distinct types of PDTO: cystic and solid, reflecting the morphological diversity typically observed in renal tumors in vivo. Cystic PDTO are characterized by central cavities surrounded by a single or multiple layers of epithelial cells. These cavities suggest a polarized cellular organization, partially mimicking the architecture of normal renal tubules. ***R24.004, R24.012, and R24.014: These solid PDTO represent dense cellular aggregates without internal cavities, reflecting disorganized growth and loss of polarity—key characteristics of tumor transformation. Necrotic areas may appear at the center of some PDTO, often due to rapid growth and insufficient nutrient supply.

Microscopic examination revealed two predominant PDTO structures: cystic and solid. Cystic organoids featured central cavities surrounded by epithelial layers, resembling renal tubules, whereas solid organoids exhibited compact, disorganized growth (Fig. 2).

The PDTO expanded over time, particularly during the first two passages. Cystic structures increased in diameter from an average of 300 µm at passage 1 to 500 µm at passage 2. Conversely, solid PDTO decreased in number with each passage, possibly due to a decrease in cell viability.

The morphological diversity observed is consistent with the known tumor heterogeneity in ccRCC. Central necrotic areas were frequently noted, likely due to rapid proliferation and limited nutrient availability.

### Immunohistochemical characterization

**Fig. 3:**
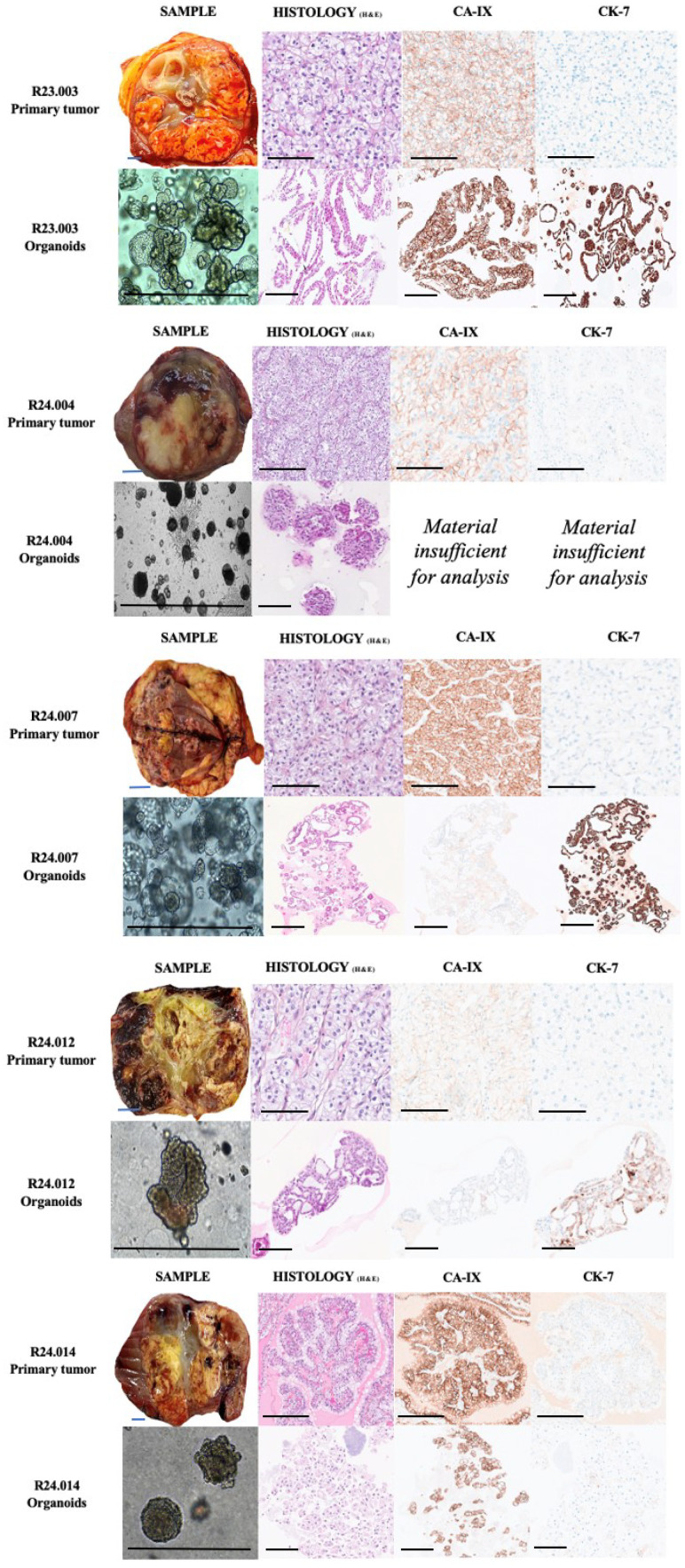
Histological and immunohistochemical characterization of ccRCC organoids (magnification *40). blue scale bar: 1 cm, black scale bar: 100 µm. R23.003: The tumor shows a disorganized architecture with high cellular density and clustered formations. Cells exhibit nuclear pleomorphism and clear or vacuolated cytoplasm, indicating glycogen or lipid accumulation. It expresses CA-IX but not CK-7. PDTO display dense nuclei, active proliferation, and glandular/tubular structures. Nuclear pleomorphism and minimal stroma confirm malignancy. CA-IX is strongly positive, while unexpected CK-7 positivity suggests culture-induced plasticity. R24.004: The tumor presents a lobular ccRCC architecture with clear cytoplasm and prominent nucleoli. It expresses CA-IX but not CK-7. PDTO form compact spheroids, reflecting clonal growth and active proliferation. Nuclei show marked heterogeneity, with some enlarged, suggesting high mitotic activity. Cytoplasmic clarity is reduced, possibly due to staining or culture conditions. Immunohistochemistry was not performed due to limited material. R24.007: The tumor exhibits a diffuse clear-cell arrangement with CA-IX positivity and CK-7 negativity. PDTO display tubular and glomeruloid structures with high cellular density and nuclear pleomorphism. Some cells show multinucleation, suggesting neoplastic activity. CA-IX expression is moderate, while strong CK-7 positivity may indicate differentiation toward another renal carcinoma subtype. R24.012: The tumor exhibits clear-cell cytoplasm, prominent nucleoli, and abundant vascularization with capillaries containing erythrocytes, indicative of angiogenesis in ccRCC. CA-IX is positive, CK-7 negative. PDTO display a disorganized architecture with tubular and alveolar structures, signs of necrosis, and nuclear pleomorphism. Weak CA-IX staining suggests moderate expression, while CK-7 is strongly expressed, suggesting dedifferentiation. R24.014: The tumor shows clear-cell proliferation arranged in trabecular and acinar structures with hyperchromatic nuclei and marked vascularization, characteristic of CA-IX is expressed, CK-7 is absent. PDTO exhibit disorganized growth, necrotic areas, and significant pleomorphism with mitotic figures. CA-IX is strongly expressed, while CK-7 expression is weak and sporadic.

Immunohistochemical (IHC) staining was performed on five of the twelve established PDTO (42%). Organoids showed morphological features broadly consistent with the corresponding primary tumors. CA-IX positivity supported their ccRCC phenotype, whereas CK7 expression varied among organoids and was sometimes discordant with the primary tumor profile (Fig. 3).

Histological analysis of primary tumor R23.003 revealed a dense cellular architecture with hyperchromatic nuclei, consistent with aggressive ccRCC features. Corresponding PDTO displayed similar morphology but showed an increase in mitotic activity. Strong CA-IX staining further confirmed their histological subtype, while CK7 expression was unexpectedly positive.

Primary tumor R24.004 displayed classic ccRCC architecture, with clear cytoplasm and prominent nucleoli. The corresponding PDTO formed compact structures, but IHC could not be performed due to limited paraffin-embedded material.

Other tumor-derived organoids showed similar variability in structure, with some expressing CA-IX strongly, while CK-7 was inconsistently expressed, possibly reflecting differentiation or plasticity in culture. Necrotic areas were commonly observed in rapidly growing PDTO, highlighting challenges in maintaining viable structures for extended periods. These results are summarized in Fig. 3.

## Discussion

Organoids have emerged as promising models for tumor biology research and personalized medicine. However, as highlighted by Ooft et al., their clinical application remains limited by the low establishment rate of PDTO [15]. This rate varies significantly by cancer type, reaching 80–90% for colorectal and pancreatic cancers [16] but dropping to approximately 20% for lung or prostate cancers [17]. In our study, the establishment rate was 42%, lower than the 67–88% reported for clear cell renal cell carcinoma (ccRCC) [12,[18], [19], [20]]. Several factors may explain this variation: (i) the relatively small tumor sizes in our cohort (50% of specimens were pT2 or smaller), (ii) variability in cell concentration due to ccRCC heterogeneity, (iii) the use of cryopreserved samples, which may have compromised cell viability, and (iv) contamination by non-tumor cells from surrounding tissues, reducing the proportion of viable tumor cells in culture [21], (v) testing several media, reduced the number of cells allocated for each condition. To improve PDTO establishment rates, refining preparation and culture conditions is essential. Optimization strategies such as enhanced dissociation methods, adapted culture supports, and techniques like red blood cell lysis—successfully applied to ccRCC spheroids [22]—could prove beneficial.

Beyond culture methodology, the primary contribution of this study lies in integrating ccRCC organoid generation within the UroCCR framework. UroCCR is a French national multicenter prospective database comprising over 20,000 patients across >60 participating centers, with standardized collection of clinical, pathological, and survival data. By linking organoid cultures to this established infrastructure, our approach provides a foundation for future ancillary studies correlating organoid-based drug sensitivity profiles or molecular features with real-world patient outcomes. The parallel biobanking strategy—including mirror samples for histology, cryopreservation, and molecular analyses—further ensures that organoid findings can be cross-validated with matched tumor tissue. This integration within a structured clinical ecosystem may ultimately facilitate the translation of organoid platforms into clinically actionable tools for personalized medicine in ccRCC.

Standardizing culture methods and selecting the optimal medium composition are crucial for improving tumoroid models [23]. Despite following multiple protocols, our study did not identify an ideal culture medium [15,19,24]. Even small variations in media composition significantly affected establishment rates depending on tumor subtypes [25], highlighting the intrinsic heterogeneity of ccRCC and the need for further refinements. While we found no significant difference between ADMEM and ADMEM/F12 media, EGF was essential due to the epithelial nature of ccRCC cells [26]. Senkowski et al. suggest using two different culture conditions to maximize the rate of establishment [27]. Apoptosis inhibitors such as Y-27,632 dihydrochloride, a ROCK-1 inhibitor, were critical for cell survival—removal after seven days, as suggested in some protocols, led to compromised cell growth [13]. Conversely, factors such as A83–01 (TGF-β inhibitor) and R-spondin1 (Wnt pathway activator) should be avoided, as they promote the proliferation of normal epithelial cells [13].

Beyond efficient PDTO establishment, optimizing the time required to obtain clinically relevant therapeutic responses is crucial. Our current protocol involves three passages, spaced 21 days apart, to ensure stable culture expansion. While this follows established recommendations [12,19,24], it significantly prolongs culture duration, thereby delaying both molecular analyses and therapeutic testing. Some studies suggest that earlier passages [20,28] may allow for faster characterization, reduce costs and accelerate drug testing—particularly for non-viable samples. Additionally, the probability of successful culture establishment declines with each additional passage. To overcome these challenges, alternative culture models such as tumor explants, or further optimization of PDTO growth conditions, could provide valuable solutions. Reducing the time required to generate clinically actionable data would facilitate the integration of PDTO into routine clinical practice, particularly for patients at risk of recurrence or metastatic progression.

Many research teams are developing miniaturized and microfluidic technologies to enable high-throughput drug testing with a limited number of PDTO. Recently, a micro-well system allowed lung tumor organoid evaluation within one week [29]. Miniaturized microfluidic devices, designed for molecular and functional analyses of individual tumor organoids, are highly promising. By providing precise control over nutrient supply, oxygenation, and waste removal, these systems improve organoid viability and reproducibility [30]. Additionally, advances in rapid imaging and automated data processing are accelerating high-throughput screening [31]. For personalized medicine applications, high-throughput procedures capable of analyzing multiple patient samples and therapeutic compounds are essential. Standardizing critical steps—such as seeding, passaging, medium changes, treatment application, biochemical assays, and 3D imaging—will be key to integrating these functional tests into clinical workflows. Ultimately, these innovations could facilitate the development of "chemograms" or "immunograms," tools that oncologists increasingly rely on for precision medicine. Thus, adjuvant immunotherapy, when indicated, should be started within three months of nephrectomy. ccRCC-derived organoids exhibit two primary morphological forms: cystic and solid [20]. Cystic organoids display rounded, thin-walled structures with a hollow center, resembling epithelial tumor rosettes, whereas solid organoids exhibit compact, disorganized multicellular growth. Li et al. attributed cystic formation to the absence of SB-202,190, a selective p38 MAP kinase inhibitor [13]. However, despite systematically incorporating SB-202,190 in our cultures, cystic PDTO persisted [20]. Quantitative growth kinetics were not formally assessed in this study; organoid expansion was monitored qualitatively by phase-contrast microscopy, with cystic structures increasing from approximately 300 µm to 500 µm between passages 1 and 2. Future studies should incorporate standardized growth curve measurements and proliferation markers such as Ki-67 to better characterize organoid viability and replicative capacity over time. Histologically, PDTO largely retained key features of the original tumor, including clear cytoplasm rich in glycogen and round nuclei with prominent nucleoli, hallmarks of ccRCC. Immunohistochemistry (IHC) confirmed the expression of tumor markers such as CA-IX and CK7. This expression is rather homogeneous in ccRCC. Interestingly, we observed variability in CA-IX and CK7 expression among some organoids, even when their corresponding primary tumors or early-stage cultures did not display these markers, suggesting potential adaptive responses to the culture environment. While ccRCC-specific markers such as PAX8, CA-IX, and CK7 are typically expressed in tumor organoids [18,20], CK7 overexpression appears more frequent in PDTO than in primary tumors [32], possibly reflecting progressive dedifferentiation after multiple passages or expansion of minor subclone. However, it should be noted that this phenomenon has been described by other teams in ccRCC although CK7 expression appeared to remain negative [33]. This phenomenon is also found in breast cancer. NOTCH inhibitor could prevent dedifferentiation [34]. Notably, our characterization occurred after six weeks in culture (three passages), whereas some studies assessed PDTO as early as 7–14 days [12,19,35]. Our immunohistochemical characterization was limited to CA-IX and CK7 due to restricted paraffin-embedded material; additional markers such as PAX-8 or CD10 would provide more comprehensive phenotypic validation and should be included in future studies with larger cohorts. These findings highlight the need for rapid, homogeneous, and faithful tumor organoid models that accurately reflect primary tumor characteristics. Achieving this will require early and repeated characterization analyses, which pose logistical challenges in terms of sample availability and culture standardization. Molecular characterization, including somatic mutation analysis and transcriptomic profiling, was not performed in this feasibility study. Previous reports have demonstrated that ccRCC organoids preserve the mutational landscape and gene expression profiles of parental tumors [33], supporting their biological relevance as preclinical models. Genomic and transcriptomic validation should be incorporated in future studies to confirm that organoids derived using our protocol faithfully recapitulate the molecular features of the original tumors. Expanding cohort sizes, increasing initial cell concentrations, and optimizing well density could enhance the feasibility of these studies.

Despite their potential, current PDTO models have significant limitations, particularly the absence of immune cells, which precludes evaluation of immunotherapy responses. This is particularly relevant in ccRCC, where therapeutic strategies target the tumor microenvironment, including immune infiltrates and the vascular system. One approach to addressing this limitation is enriching PDTO with autologous stromal cells. Recent efforts have focused on integrating microenvironmental components through direct co-cultures or sophisticated microfluidic systems. For instance, PDTO can be co-cultured with T lymphocytes derived from PBMCs or intra-tumoral lymphocytes to assess immune checkpoint inhibitor efficacy, such as PD-1, PD-L1, or CTLA-4 [36,37]. In this protocol, despite the presence of stromal components and extracellular matrix, no further analysis of the tumor microenvironment was performed. The air-liquid interface technique also preserves immune infiltrates but requires specialized and costly equipment [38]. Our current model also lacks a vascular system, limiting its utility in evaluating responses to anti-angiogenic tyrosine kinase inhibitors. Potential solutions include integrating microfluidic systems for optimized gas exchange or co-culturing with endothelial cells [6,39]. However, due to insufficient PDTO growth in our study, we were unable to conduct the necessary analyses to characterize these microenvironmental elements. Future research should focus on refining these models to enhance their reliability and reproducibility. Advances in co-culture platforms, such as organoid-on-chip systems, hold promise for evaluating PDTO responses across a broad spectrum of therapeutic strategies, particularly those targeting the tumor microenvironment. As these technologies mature, tumor PDTO are poised to transition from experimental tools to essential components of precision oncology, ultimately improving treatment outcomes for patients.

## Conclusion

ccRCC organoids offer a promising platform for personalized medicine, enabling patient-specific treatment strategies. However, several challenges must be addressed to optimize their clinical applicability. Improving establishment rates, refining culture conditions, and ensuring that PDTO faithfully recapitulate the original tumor are key priorities. Integrating stromal and immune components may enhance their predictive accuracy for therapeutic responses, while high-throughput methodologies may facilitate large-scale applications. Overcoming these challenges is essential to transition ccRCC organoids from experimental models to clinically relevant tools, ultimately contributing to biomarker discovery, drug testing, and precision oncology advancements.

## Author ethics statement

All authors have no conflicts of interest to disclose.

## Financial support

French Urological Association (AFU) with IPSEN grant, Cancéropôle Nord Ouest (CNO): High Risk High Potential N°2023/13 grant, Université de Caen-Normandie and CNRS

## CRediT authorship contribution statement

**R. Lefranc:** Writing – original draft, Methodology, Formal analysis, Data curation, Conceptualization. **T. Waeckel:** Writing – review & editing, Writing – original draft, Resources, Project administration, Funding acquisition, Formal analysis, Data curation. **M. Riffet:** Investigation, Data curation. **R. Florent:** Writing – review & editing, Supervision, Resources, Methodology, Investigation, Data curation, Conceptualization. **G. Desmartin:** Supervision, Resources, Methodology, Conceptualization. **L. Lecouflet:** Supervision, Methodology. **J. Divoux:** Supervision, Methodology. **L. Poulain:** Methodology, Conceptualization. **X. Tillou:** Supervision, Project administration, Investigation, Funding acquisition. **L.B. Weiswald:** Writing – review & editing, Validation, Supervision, Project administration, Methodology, Data curation, Conceptualization. **G. Levallet:** Writing – review & editing, Validation, Project administration, Methodology, Funding acquisition, Conceptualization. **C. Bazille:** Writing – review & editing, Validation, Supervision, Project administration, Methodology, Funding acquisition, Conceptualization.

## Declaration of competing interest

The authors declare that they have no known competing financial interests or personal relationships that could have appeared to influence the work reported in this paper.
